# Managing chronic pathologies with a stepped mHealth-based approach in clinical psychology and medicine

**DOI:** 10.3389/fpsyg.2015.00407

**Published:** 2015-04-14

**Authors:** Gianluca Castelnuovo, Italo Zoppis, Eugenio Santoro, Martina Ceccarini, Giada Pietrabissa, Gian Mauro Manzoni, Stefania Corti, Maria Borrello, Emanuele Maria Giusti, Roberto Cattivelli, Anna Melesi, Giancarlo Mauri, Enrico Molinari, Francesco Sicurello

**Affiliations:** ^1^Istituto Auxologico Italiano IRCCS, Psychology Research Laboratory, Ospedale San GiuseppeVerbania, Italy; ^2^Department of Psychology, Catholic University of MilanMilan, Italy; ^3^Department of Informatics, Systems and Communication, Università degli Studi di Milano-BicoccaMilano, Italy; ^4^Laboratory of Medical Informatics, Department of Epidemiology, Istituto di Ricovero e Cura a Carattere Scientifico – Istituto di Ricerche Farmacologiche Mario NegriMilano, Italy; ^5^Department of Psychology, University of BergamoBergamo, Italy; ^6^Department of Electronics, Information and Bioengineering, Politecnico di MilanoMilano, Italy

**Keywords:** chronic care management, mHealth, e-health, clinical psychology, new technologies, rehabilitation, behavioral medicine, health psychology

## Abstract

Chronic diseases and conditions typically require long-term monitoring and treatment protocols both in traditional settings and in out-patient frameworks. The economic burden of chronic conditions is a key challenge and new and mobile technologies could offer good solutions. mHealth could be considered an evolution of eHealth and could be defined as the practice of medicine and public health supported by mobile communication devices. mHealth approach could overcome limitations linked with the traditional, restricted, and highly expensive in-patient treatment of many chronic pathologies. Possible applications include stepped mHealth approach, where patients can be monitored and treated in their everyday contexts. Unfortunately, many barriers for the spread of mHealth are still present. Due the significant impact of psychosocial factors on disease evolution, psychotherapies have to be included into the chronic disease protocols. Existing psychological theories of health behavior change have to be adapted to the new technological contexts and requirements. In conclusion, clinical psychology and medicine have to face the “chronic care management” challenge in both traditional and mHealth settings.

## The Need of a Chronic Care Management Approach for the Long-Term Treatment of Many Pathologies

Chronic diseases and conditions (cardiovascular pathologies, diabetes, obesity, COPD-chronic obstructive pulmonary disease, chronic pain, traumatic brain injuries, etc.) more common in elderly persons, typically are requiring long-term monitoring and treatment protocols both in traditional settings and in out-patient frameworks. Significant increases in managing this category of patients are due to clinical improvements, better screenings, and reliable diagnoses of medical and psychological pathologies that enable those with chronic conditions to live longer. Anyhow, clinically and cost effective management of such conditions is increasingly required in order to ensure a sustainable health care system.

In fact, treating these chronic diseases cost billions of dollars each year within the US (Weingarten et al., 2002; National Center for Health Statistics, 2009; Mercer, 2011). The healthcare system is suffering a long period of crisis in North America (O’Donohue et al., 2009) and “Most view healthcare as too costly, of uneven quality, difficult to access, and inefficient. Behavioral healthcare is no different” (O’Donohue and Draper, 2011a, p. 1). The economic burden of chronic conditions is a key challenge and only in the US approximately 125 million individuals have at least one chronic condition (an estimated 157 million in 2020), with half of this population suffering from more than one condition (Wu and Green, 2000).

Disease management, defined as an integrated coordination of healthcare interventions and actions for populations with chronic conditions, is a possible solution to these growing healthcare costs. According to Mercer (2011), disease management “supports the physician or practitioner/patient relationship and plan of care; emphasizes prevention of exacerbations and complications through the use of evidence-based practice guidelines and patient empowerment strategies; and evaluates clinical, humanistic, and economic outcomes on an ongoing basis with the goal of improving overall health” (Mercer, 2011, p. 152). Another interesting approach and solution is the Chronic Care Model, developed by Wagner et al. (2001a,b; Glasgow et al., 2001), that is based on the collaboration between a well coordinated team of clinicians-providers and an actively engaged patient, promoting self-management skills, tracking, and sharing information about patient health status and treatment programs, focusing on the family, social, and community networks (O’Donnell, 2011).

In the management of chronic diseases clinical psychology plays a key role (Castelnuovo, 2010a,b), due to the need of working on psychological conditions of patients, their families and their caregivers (Levy et al., 2007; de Ridder et al., 2008; Pagnini et al., 2010, 2011, 2012), particularly with cardiovascular diseases where psychological variables (anxiety, stress, depression, etc.) have a significant impact in the organic worsening and demanding caregiving with developed case management skills is requested, even if relatives and caregivers are not well trained in accomplishing healthcare tasks (Hemingway and Marmot, 1999a,b; Rozanski et al., 1999; Manzoni et al., 2011a). About chronic disease management programs that focus on containing costs and improving health outcomes (Villagra, 2004; Villagra and Ahmed, 2004), Mercer (2011, p. 151) noted that “What emerged from these early programs was an understanding that quality improvement and cost reductions could be achieved through enhancing disease process understanding and attending to the psychological aspects of health and illness (Schneiderman et al., 2001; Levy et al., 2007).”

## Opportunities Provided by a Stepped mHealth-Based Approach in the Chronic Care Management

There is a growing interest in using new and mobile technologies for the enhancement of chronic disease self-management, generally including symptom monitoring, medication adherence, patient education for improving healthier lifestyles (diet, physical activity, etc.; Spruijt-Metz et al., 2015).

eHealth could be traditionally defined as a growing field of health services provided through the Internet and other new technologies (Eysenbach, 2001).

mHealth (also m-health, mHealth, or mobile health) could be considered an evolution of eHealth and could be defined as the practice of medicine and public health supported by mobile communication devices, such as mobile phones, tablet computers, and PDAs, for health services and information (Riper et al., 2010; Eysenbach, 2011; Cipresso et al., 2012; Whittaker, 2012; Fiordelli et al., 2013; Castelnuovo et al., 2014). Another interesting definition of mHealth, provided in an engineering field, defines it as “the practice of eHealth supported by mobile devices and smartphones, which are used to capture, analyze, store, and transmit health-related information from various sources including personal inputs, sensors, and other biomedical acquisition systems” (Adibi, 2015, p. 2).

mHealth approach could overcome limitations linked with the traditional, restricted and highly expensive in-patient treatment of many chronic pathologies: one of the best up-to-date application is the management of obesity with type 2 diabetes, where mHealth solutions can provide remote opportunities for enhancing weight reduction and reducing complications from clinical, organizational, and economic perspectives (Manzoni et al., 2008, 2011b; Khaylis et al., 2010; Rao et al., 2011). Specifically for diabetes management Chomutare et al. (2011) reported more than 260 different diabetes applications (for Nokia Symbian, BlackBerry, Apple iPhone, and Google Android), able to manage many features of the diabetes management, self-monitoring, blood glucose, weight, physical activity, diet, insulin and medication, blood pressure, education, disease-related alerts and reminders, integration of social media functions (Santoro et al., 2011; Santoro, 2013; Santoro and Quintaliani, 2013), disease-related data export and communication, synchronization with personal health record (PHR) systems, and patient portals (Chomutare et al., 2011).

Levy (2012) noted that, “Mobile healthcare (mHealth) is the biggest technology breakthrough of our time (being used) to address our greatest national challenge,” and worldwide “the technology and its promise have moved up the healthcare agenda,” said US Health and Human Services Secretary, Kathleen Sebelius, in her keynote address at the 2011 mHealth summit held in the Washington DC area (Levy, 2012, p. 3). The new technologies behind the mHealth approach are moving from interesting but isolated applications (i.e., apps), toward a single patient-tailored, engaging, preventing, monitoring, treating, and less expensive health care system. Moreover, industry reports that mHealth is representing a significant emerging development in globalized health care markets (Levy, 2012; West, 2012; Malvey and Slovensky, 2014).

In a well structured and stepped mHealth approach, patients with chronic diseases usually interact with caregivers any time as soon as symptoms appears avoiding useless visits in hospital; physicians communicate with patient obtaining continuous information from biometric sensors avoiding hands-on examinations where no necessary. About taking medications, a typical chronic care scenario, periodically text, or vocal reminders will ensure patient compliance in taking medicines as prescribed by physicians avoiding unnecessary hands-on examinations. Moreover, patients can be monitored and treated in their everyday contexts, following the approach “move the healthcare where it really needs” (Castelnuovo, 2008; Castelnuovo et al., 2010, 2011): in traditional context clinicians can monitor in a discontinuous setting, whereas in a mHealth approach the disappearing (not invasive) but continuous monitoring allow patients to receive much more health messages and feedback avoiding a coming back into unhealthy lifestyle conditions or behaviors. The new approach does not substitute the old one but integrates it: if the remote monitoring will indicate a worsening of clinical conditions or parameters, a traditional approach (in-patient visit, hospitalization, etc.) will be used (Mutingi, 2015).

mHealth delivery innovations could be implemented in many health care categories, such as communication between patients and health service providers (health call centers, emergency telephone services, appointment reminders, treatment compliance, etc.), community health promotion, discussion between different health care professionals, managing emergencies, continuous health monitoring-surveillance, etc. (World Health Organization, 2011; Malvey and Slovensky, 2014).

It is also interesting to note that mHealth is creating new challenges from a different theoretical perspective: the need to develop new theoretical models and methods for both integrating heterogeneous sources of data (Cava et al., 2013, 2014) and analyzing huge amount of (relational) information (Zoppis et al., 2007; Zoppis and Mauri, 2008) which can be collected by remote devices.

Working out the previous Von Korff and Tiemans’s (2000) proposal, a strong model of stepped care based on mHealth is proposed by O’Donnell (2011, p. 265): “the stepped care model is based on the acknowledgment that (1) different patients require different levels of care; (2) the most appropriate level of care is based on closely monitoring outcomes; and (3) moving from lower to more intensive levels of care based on patient response can increase the effectiveness of care while lowering overall costs.” Stepped care is “potentially much more consistent with the ethical imperative of choosing the least intrusive intervention for one’s patient” (O’Donohue and Draper, 2011a, p. 3). Using this approach, many efforts in the research field have to be focused not only in the development of new clinical protocols or therapies, but in the validation of new health-care delivery model, measuring its reliability, affordability, safety, efficiency, and user satisfaction (where users are patients, professionals, stakeholders, etc.) and demonstrating that this model can improve the quality of care reduce costs (Weingarten et al., 2002; Neumeyer-Gromen et al., 2004; Ofman et al., 2004; Mercer, 2011; O’Donohue and Draper, 2011a).

In the pioneering book *Stepped Care and e-Health Practical Applications to Behavioral Disorders*, O’Donohue and Draper (2011b, p. 5–6) proposed a practical stepped-care model for many pathologies, including chronic conditions. New technologies play an important role in this model (point 5), even if mHealth does not express all its potentiality. A list of the health care “steps” is indicated below:

(1) Assessment and Triage...
(2) Watchful Waiting...
(3) Psychoeducation...
(4) Bibliotherapy...
(5) E-Health...
(6) Group Therapy...
(7) Individual Therapy...
(8) Medical and Medication...
(9) Inpatient Treatment...

Each disease management program, including or not a mHealth stepped approach, has to be evaluated in relationship to cost issues, such as the measurement of return on investment (ROI; O’Donnell, 2011). Some evidences about pros and cons of this approach are now available in scientific literature: one of the best review (Mattke et al., 2007) evaluated different types of disease management programs about quality, health outcomes and cost for various chronic conditions (three large-scale population-based studies, 10 meta-analyses, 16 systematic reviews containing 317 unique studies were considered). The article noted that there was significant evidence that disease management improves the processes of care allowing a more functional disease control, but no important clinical evidences were found in long-term periods (perhaps for lacking follow-ups). Moreover, no conclusive results were found about cost savings (O’Donnell, 2011).

Possible guidelines for a mHealth economic evaluation have been provided by Kahn et al. (2010) and reported in Malvey and Slovensky (2014, p. 153):

• “Description of the mHealth intervention
• Computed costs of the intervention
• Potential drawbacks and adverse effects of using this intervention versus another or none
• Awareness of practical/real-world issues such as sustainability of the product, costs, and outcomes.”

Other economic evaluation methods are available for evaluating mHealth technologies such as contingent valuation analysis (CVA), conjoint analysis (CA), comparative effectiveness research (CER), cost-effectiveness analysis (CEA), cost utility analysis (CUA), and cost-minimization analysis (CMA; Martin and Solano, 2015).

Unfortunately, many barriers for the spread of mHealth are still present (Gaggioli et al., 2005; Rees and Stone, 2005) and are well summarized by Mohammadzadeh and Safdari (2014): organizational and technological barriers; negative user attitudes; difficult technology acceptance; threats to confidentiality and privacy; legal, ethical, and administrative barriers; high costs of system implementation and system maintenance; lack of sufficient investment; poor design and implementation; lack of system interoperability with electronic health records and other IT tools; poor functioning of system that leads to medical errors and negative effects on care outcomes, patients, and personnel; mistakes in documentation; data manipulation and violation of patients’ legal rights; sudden interruptions of telecommunication networks.

## Focusing on Psychosocial and Behavioral Determinants in the mHealth-Based Approach for the Chronic Care Management

In mHealth stepped chronic care management psychosocial and behavioral aspects have to be considered (Schneiderman et al., 2001). Due the significant impact of these factors on disease evolution, psychological interventions, and psychotherapies have to be included within the chronic disease protocols (Castelnuovo et al., 2003; Cummings and Cummings, 2005; Castelnuovo, 2010a,b), trying to transform a “daily care for (chronic) patients from treatment that is acute and reactive, to treatment that is proactive, planned, and population based” (Coleman et al., 2009; Mercer, 2011, p. 153). The goals of complex chronic disease management are developing an integrated and effective team care and supporting self-management resources involving family and community members for each patient (Coleman et al., 2009; Mercer, 2011). An attitude of patient engagement and patient empowerment is necessary for a reliable long-term chronic care model (Buccoliero et al., 2010; Barello et al., 2012; Graffigna et al., 2013a,b, 2014)

Wagner et al. (1996, p. 514) noted that many risks of failure in managing chronic disease patients are connected to psychological variables:

“(1) Delays in the detection of complications or declines in health status because of irregular or incomplete assessments or inadequate follow-up; (2) Failures in self-management of the illness or risk factors as a result of patient passivity or ignorance stemming from inadequate or inconsistent patient assessment, education, motivation, and feedback; (3) Reduced quality of care due to the omission of effective interventions or the commission of ineffective ones; (4) Undetected or inadequately managed psychosocial distress.”

Existing psychological theories of health behavior change have to be adapted to the new technological contexts and requirements:

“to fully leverage the potential of mobile technologies for health behavior interventions, health behavior theories need to be able to guide the development of complex interventions that adapt rapidly over time in response to real-time and real-world inputs. As intervention developers take full advantage of mobile technologies, health behavior models will be required to guide tailored adjustments not only at the start of an intervention but also through the dynamic process of frequent iterative adjustments during the course of intervention. The content and timing of a specific intervention can be driven by a range of variables including (1) the target behavior frequency, duration, or intensity; (2) the effect of prior intervention effects on the target behavior; and (3) the current context of the individual. Such interventions require health behavior models that have dynamic, regulatory system components to guide rapid intervention adaptation based on the individual’s current and past behavior and situational context”

(Spruijt-Metz et al., 2015, p. 127).

In clinical health psychology different methods have been developed to enhance health behavior change: ((s))Prochaska and DiClemente (1992) transtheoretical stages of change model (TTM; Riemsma et al., 2002), Hochbaum and Rosenstock’s health belief model (Green et al., 1994), Bandura’s (1977, 2004) and Bandura et al. (1977) self-efficacy theory, Gabrielsen’s concept of action competence (Larsen and Zwisler, 2004). Particularly, a growing approach in chronic care management is represented by Motivational Interviewing (Bellg, 2003; Brennan et al., 2008; Everett et al., 2008; Miller, 2010, 2012; Beckie and Beckstead, 2011; Bredie et al., 2011), a client-centered yet directive method for enhancing intrinsic motivation to change by exploring and resolving client ambivalence (Burke et al., 1997; Miller et al., 1997).

Khaylis et al. (2010) underlined five psychological features necessary for a positive technology-based and mHealth-based chronic care management in obesity and weight-loss: (1) *self-monitoring* (patients monitoring and regulating their own behaviors); (2) *counselor feedback and communication* (clinicians motivating and encouraging patients in achieving healthier lifestyles); (3) *social support* (group treatments favoring improvements); (4) *Structured program* (stepped protocols including regular interventions on different areas such as eating, physical activities, coping strategies and problem-solving); (5) *Individually tailored program*: (creating customized interventions according to patients’ resources and needs).

## Future Trends for a Successful Spreading of the mHealth-Based Approach in Chronic Care Management

The emergence and spread of an “apps” culture is a current reality: the new normal mode is to access the Internet via cell phones, whereas laptops and desktop computers were the standards in the past (Purcell et al., 2010). Moreover, many future patients prefer virtual visits in comparison with traditional ones. “A major study by Cisco found that fully 74% of consumers are open to virtual doctor visits” using technology to improve access and convenience, especially when the e-visit with an online physician is followed by a telephone or e-mail “check-in” a few days later to see how the patient is feeling” (Malvey and Slovensky, 2014, p. 30). Even if strategic utilization of mHealth products received much attention in health-care industry and consumers (Pak and Park, 2012; Shin, 2012), academic research is lacking.

However, some key questions, well described by Malvey and Slovensky (2014, p. 180) are necessary in order to ensure a reliable business model in the mHealth field: “in this type of environment, investment decision making is complicated by high levels of uncertainty, with concerns focused on:

• Will the product work as intended?
• What is the probability of long-term adoption?
• Can the product be developed and implemented at a market-competitive price?
• Will the product be easy to replicate or supplant by competitors?
• Will continuing product revisions be required?
• Will the product confront unanticipated legal or regulatory challenges?”

Malvey and Slovensky (2014, pp. 189–190) noted that specific factors are key elements in order to obtain a successful mHealth care delivery system:

• “*Establishing and assuring both privacy and security of data transmission*. There is no compromise on this point for either consumers or providers of health care...
• *Creating a mHealth certification program that works is a priority*...
• *Eliminating regulatory uncertainty is requisite for mHealth to progress*...
• *Producing rigorous evidence showing that mHealth has an impact on health, access to care, cost, quality, and patient satisfaction is essential*. Up until now, everyone, including investors, providers, consumers, and governmental entities, has taken the benefits of mHealth on faith; that is, they assumed that mHealth was having a positive impact. To move forward, these stakeholders require confirmation that mHealth is achieving its intended goals. Establishing payment or reimbursement models for mHealth is essential...
• *Focusing on the workflows rather than the gadgets is imperative*, because the goal is to improve clinical and non-clinical workflows through mobile technologies...
• *Developing apps that focus on the end user is critical and is necessary to assure a promising future for adoption*. Apps that are difficult to use can lead to non-use...Reasons for non-use include the fact that many of the apps still require manual inputting of data; have problems integrating with existing blood-glucose meters; or simply fail to measure blood sugar, activity, and food intake adequately...
• *Achieving sustainability, financial stability, and diffusion of technology requires establishing actionable goals for developers, entrepreneurs, and innovators, as well as payers, policymakers and others* who view mHealth as essential to revolutionize health delivery systems.”

In conclusion, clinical psychology and medicine have to face the “chronic care management” challenge in both traditional and mHealth settings, providing more evidence-based protocols and organizational models. Psychological interventions have already demonstrated their clinical effectiveness and the future focus will be mainly on providing cost-utility evidences, persuading stakeholders that a health system with clinical psychology is clinically better and economically cheaper than a health system without psychological interventions. A stepped-care approach could better show how cost-savings are possible. The mHealth scenario could help clinicians in managing chronic situations through the possibility of monitoring each organic and psychological conditions with many sensors that can send intelligent alerts in case of need, avoiding useless traditional visits, and reducing direct and indirect costs. In a stepped scenario, minimal treatments can provide a significant health gain, whereas more intensive and expensive medical and psychological interventions are dedicated to persons who did not benefit from simpler (first-line) treatments (Bower and Gilbody, 2005). Future research has to compare traditional models of providing health care with stepped mHealth based approaches. Unfortunately only few studies have supported the stepped care approaches in psychological interventions.

## Conflict of Interest Statement

The authors declare that the research was conducted in the absence of any commercial or financial relationships that could be construed as a potential conflict of interest.
